# PA-X 122V broadly determines the host shutoff activity of influenza A viruses

**DOI:** 10.1128/mbio.03433-25

**Published:** 2025-12-30

**Authors:** Yuying Yang, Mengmeng Xu, Naixin Zhang, Qinhao Yu, Yunfei Wan, Chengzhi Xu, Yunpu Wu, Fei Meng, Yan Chen, Huanliang Yang, Guohua Deng, Jianzhong Shi, Li Jiang, Chuanling Qiao, Hualan Chen

**Affiliations:** 1 State Key Laboratory of Animal Disease Control and Prevention, Harbin Veterinary Research Institute, Chinese Academy of Agricultural Sciences 687216 , Harbin, Heilongjiang, China; Huazhong Agricultural University, Wuhan, Hubei, China

**Keywords:** influenza A virus, PA-X protein, host shutoff, innate immune response

## Abstract

Multiple genes are involved in the pathogenicity of influenza A virus. Our previous study reported two naturally occurring amino acid mutations in the polymerase acidic (PA) protein as crucial determinants of the virulence of Eurasian avian-like H1N1 (EA H1N1) influenza viruses. PA-X, an accessory protein encoded by the PA gene, is thought to play a role in viral pathogenicity and regulation of host immune response, but its specific function remains unclear. In this study, we found that two genetically similar EA H1N1 influenza viruses, A/swine/Liaoning/FX38/2017 (FX38) and A/swine/Liaoning/SY72/2018 (SY72), induced significantly different suppression levels of host protein synthesis. The difference in host shutoff activity induced by PA-X protein was the key factor affecting the inhibition of host gene expression. Loss of PA-X expression significantly reduced its host shutoff activity, thereby enhancing host antiviral immune response. PA-X deficiency had no apparent effect on polymerase activity or replication capacity. We pinpointed a single residue 122V involved in the ability of PA-X to inhibit host gene expression and thereby modulate the host antiviral response. Notably, PA-X 122V was highly conserved among multiple subtypes of influenza A viruses and vital for maintaining the inhibitory effects on the host protein synthesis. Together, these findings demonstrate that the PA-X protein plays a major role in the suppression of host protein synthesis during influenza virus infection and elucidate the molecular mechanism by which the amino acid residue 122V in PA-X facilitates its suppression effects on host innate immune responses.

**IMPORTANCE:**

PA gene, encoding PA protein and several accessory proteins including PA-X, PA-N155, and PA-N182, is a key factor determining the pathogenicity of influenza A virus. In this study, we found that PA-X is crucial for suppression of host protein synthesis during viral infection. Loss of PA-X expression significantly reduced its host shutoff activity, thereby enhancing host antiviral immune responses. Furthermore, we pinpointed a crucial amino acid, 122V, involved in the host shutoff activity of PA-X and found that 122V is highly conserved among multiple subtypes of influenza A viruses. These findings deepen our understanding of the mechanisms by which PA-X modulates viral pathogenesis and the host immune response.

## INTRODUCTION

Influenza A virus is an enveloped, segmented, negative-sense ribonucleic acid (RNA) virus that belongs to *Orthomyxoviridae* family. It is found to infect multiple animal species, including both wild and domestic birds, as well as several mammalian species, including humans, pigs, horses, dogs, cats, and, most recently, minks and dairy cattle (1–3). The genome of influenza virus is composed of eight single-strand RNA fragments, encoding at least 10 viral proteins, including polymerase basic protein 2 (PB2), polymerase basic protein 1 (PB1), polymerase acidic protein (PA), hemagglutinin (HA), nucleoprotein (NP), neuraminidase (NA), matrix protein 1 (M1), matrix protein 2 (M2), nonstructural protein 1 (NS1), and nonstructural protein 2 (NS2) (4, 5). In addition, several new proteins, such as PB2-S1, PB1-F2, PB1-N40, PA-X, PA-N155, PA-N182, M42, and NS3, were recently discovered (6–12). PA-X is encoded by extending the ribosomal frameshift of the PA polypeptide +1 open reading frame (X-ORF) (9). Therefore, PA-X possesses the same 191 N-terminal amino acids as the PA protein but has a unique C-terminal region derived from the +1 frameshift during translation. It contains 41 or 61 highly conserved amino acids and can be detected in different hosts and subtypes of influenza viruses (13–15).

The pathogenicity of influenza A virus is determined by multiple viral and host factors. The gradual acquisition of amino acid changes in several viral proteins contributes to the virulence of influenza viruses (16–21). Viruses employ multiple strategies to block host antiviral defenses. In many viruses, this inhibition is partly achieved through a blockade of host gene expression, a process termed “host shutoff”. To counter host antiviral activity, influenza viruses possess different inhibition mechanisms, including blocking host gene expression mediated by viral NS1 and PA-X proteins (22, 23). As a host shutoff protein and the major viral inhibitor of interferon (IFN) responses, NS1 can interfere with the detection of the double-stranded RNA binding domain by host sensors and also interferes with general host gene expression by blocking nuclear processing, polyadenylation, and export of messenger RNA (mRNA), respectively (24–26). PA-X decreases host and viral mRNA accumulation via its shutoff activity, which is dependent on its endonuclease activity (27, 28). Although PA-X is not essential for viral replication *in vitro,* it modulates the host immune response *in vivo*. This host shutoff activity allows influenza virus to evade immune defenses that normally limit viral replication and spread (14, 29).

Our previous study compared the pathogenicities of two genetically similar Eurasian avian-like H1N1 (EA H1N1) influenza viruses, A/swine/Liaoning/FX38/2017 (FX38) and A/swine/Liaoning/SY72/2018, and pinpointed two amino acid residues 100 and 122 in PA as crucial determinants of their virulence differences in mice (30). In this study, we used a deficient expression of PA-X protein in the background of SY72 and FX38 to investigate the impacts of PA-X on viral replication, pathogenicity, and host immune responses. Furthermore, we identified a key amino acid 122V in PA-X involved in their host shutoff activity differences and also revealed the underlying mechanisms.

## RESULTS

### PA-X downregulates cellular protein synthesis and is a major contributor to host shutoff activity during influenza virus infection

**Fig 1:**
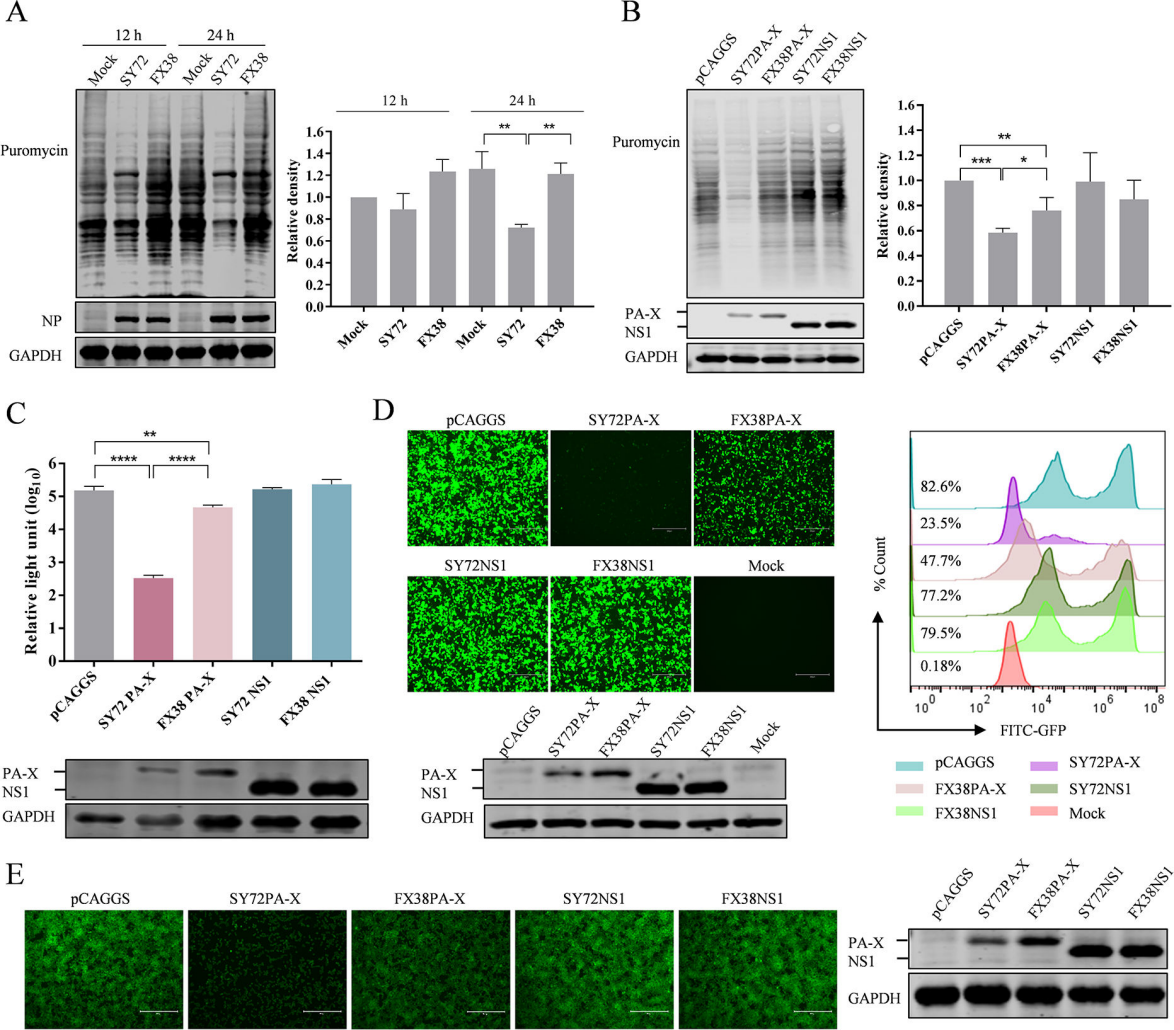
The PA-X proteins of SY72 and FX38 viruses showed different host shutoff activities. (**A**) The host shutoff activities induced by SY72 or FX38 virus. The ribopuromycylation assay was conducted in A549 cells infected with SY72 and FX38 virus at an MOI of 1, followed by addition of puromycin for 45 min. Then, the cell lysates were analyzed by western blot to detect viral NP, GAPDH, and puromycin for evaluating nascent host proteins (left panel). Uninfected A549 cells were used as a mock. The relative density of nascently synthesized proteins was quantified using ImageJ software (https://imagej.net.ij/) (right panel). The level was normalized to the mock (12 h). (**B**) The host shutoff activities induced by the PA-X and NS1 proteins of SY72 or FX38 virus. HEK293T cells were transfected with the recombinant pCAGGS plasmid expressing PA-X or NS1 for 24 h, and the global efficiency of cellular protein synthesis was then monitored by a ribopuromycylation assay (left panel). The relative density was measured as described in the Fig. 1A legend (right panel). (**C**) Inhibition of the PA-X and NS1 protein on Rluc expression. HEK293T cells were co-transfected with the plasmid pRL-TK, and pCAGGS-PA-X or pCAGGS-NS1. Cells transfected with empty pCAGGS plasmid were included as a control, and Rluc activity was normalized to the control. (**D**) The impact of PA-X and NS1 host shutoff activities on GFP expression in HEK293T cells. Cells were co-transfected with the pCAGGS plasmid encoding PA-X or NS1 of SY72 and FX38, together with the pCAGGS plasmid encoding GFP. At 24 hpt, GFP was observed under a fluorescence microscope, and fluorescent intensity was quantified using flow cytometry. (**E**) The impact of PA-X and NS1 host shutoff activities on GFP expression in HEK293T-EGFP cells. Cells were transfected with the pCAGGS plasmid encoding PA-X or NS1 of SY72 and FX38 and observed under a fluorescence microscope at 24 hpt (left panel). Scale bar, 300 μm. The presence of PA-X, NS1, and GAPDH in cells was analyzed by western blot using anti-PA-X, anti-NS1, and anti-GAPDH antibodies, respectively (right panel). The results are shown as the mean ± standard deviation (SD). *, *P* < 0.05; **, *P* < 0.01; ***, *P* < 0.001; ****, *P* < 0.0001.

Our previous study found that two genetically similar viruses (FX38 and SY72) exhibited significantly different virulence in mice (30). Here, to investigate the host shutoff activity induced by these two viruses, A549 cells were infected with the SY72 and FX38 viruses at an MOI of 1. At 12 and 24 hpi, the newly synthesized proteins were examined using a ribopuromycylation assay. As shown in Fig. 1A, a broad-spectrum inhibitory activity on cellular protein synthesis was induced by SY72 virus infection, and this activity depended on the duration of viral infection and virus accumulation. Compared to the SY72 virus, the FX38 virus did not induce detectable host shutoff activity at the two time points.

PA-X and NS1 proteins of influenza viruses were reported to induce host shutoff activity during viral infection and thereby manipulate the innate immune response (31, 32). Here, to elucidate the roles of these proteins in determining the different shutoff activities triggered by viral infection, HEK293T cells were transfected with the recombinant pCAGGS plasmids expressing the PA-X and NS1 proteins. The host shutoff activities induced by these two proteins were detected by a ribopuromycylation assay. As shown in Fig. 1B, both the SY72PA-X and FX38PA-X proteins exhibited a high level of shutoff activity, whereas no detectable shutoff activity was induced by the SY72NS1 and FX38NS1 proteins. Furthermore, SY72PA-X induced significantly stronger shutoff activity than FX38PA-X.

To investigate the inhibition efficiency of the PA-X and NS1 proteins on the expression of exogenous proteins in cells, HEK293T cells were co-transfected with a plasmid expressing *Renilla* luciferase (Rluc) or GFP together with a plasmid encoding the PA-X or NS1 gene of the SY72 and FX38 viruses, respectively. Rluc and GFP expression was then measured by a luciferase assay and flow cytometry, respectively. As shown in Fig. 1C, Rluc expression levels were significantly reduced in the presence of PA-X of SY72 and FX38, with approximately 0.21% and 28.79% of that of the control, respectively. By contrast, the expression level of Rluc was not apparently affected by the NS1 protein of SY72 or FX38. Similarly, GFP expression was significantly inhibited by PA-X, but not by NS1 of SY72 and FX38. Moreover, SY72PA-X exhibited significantly stronger inhibition of GFP expression compared to FX38PA-X (Fig. 1D). Meanwhile, we further investigated the inhibitory effect of PA-X and NS1 on GFP expression in HEK293T-EGFP cells. As shown in Fig. 1E, similar to what was observed in HEK293T cells transiently transfected with GFP, we found that PA-X, particularly SY72PA-X, exhibited a strong inhibitory effect, whereas the NS1 protein had no effect on GFP expression. Taken together, these results indicated that the FX38 and SY72 viruses differ in their host shutoff activity, and PA-X plays a key role in viral inhibition of host protein synthesis efficiency.

### PA-X deficiency significantly decreases host shutoff activity

**Fig 2:**
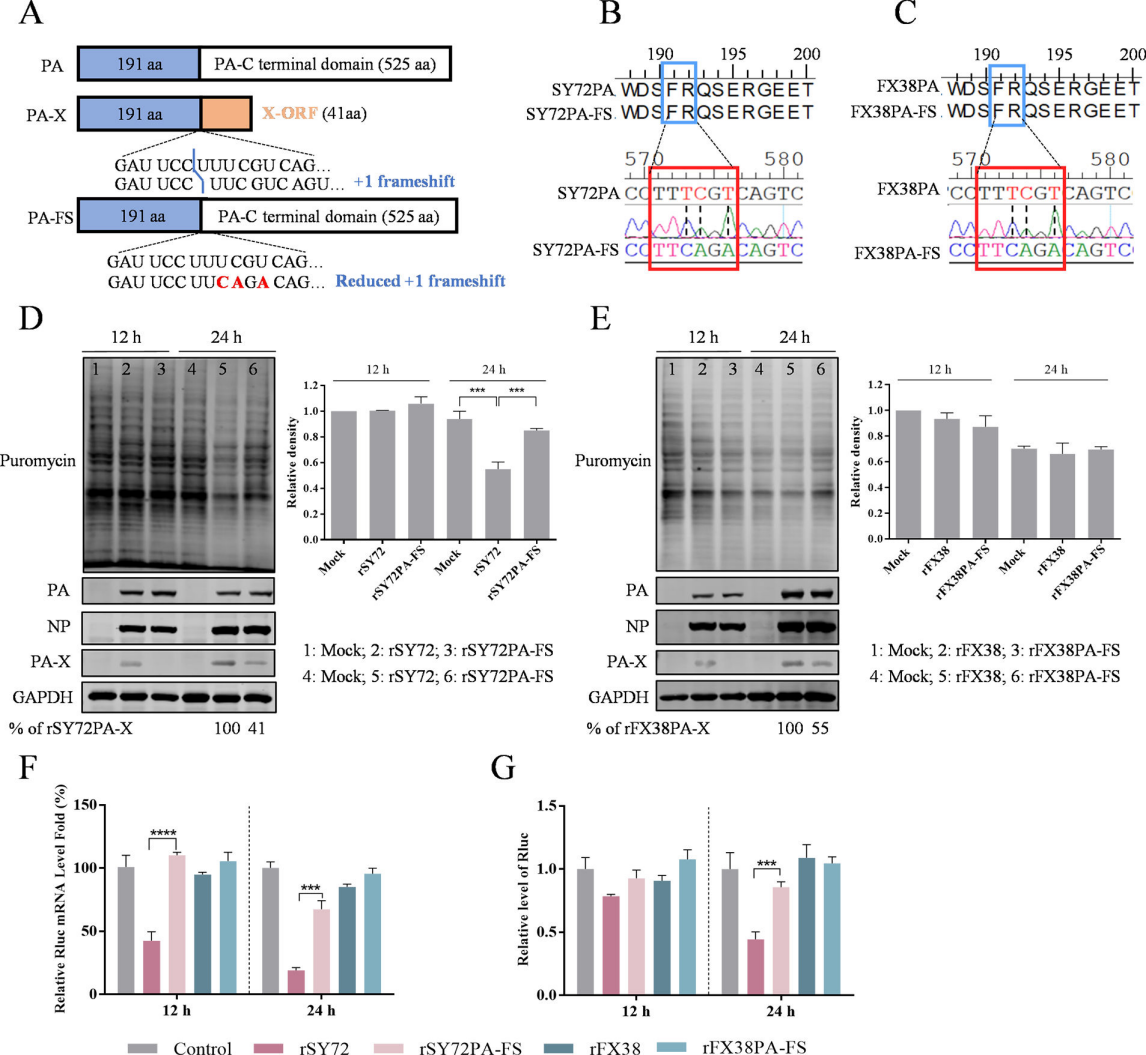
Comparison of the host shutoff activity induced by the parental SY72 or FX38 viruses, and their PA-X deficient viruses. (**A**) Schematic representation of PA and PA-X and three nucleotide mutations (indicated in red) introduced into the frameshift (FS) motif to inhibit PA-X synthesis. (**B, C**) Sequencing map of rSY72 and rFX38 PA protein mutations. The amino acids that remained unchanged following the introduction of nonsense mutations into the frameshift motif are highlighted in a blue box. Red box represents the corresponding PA coding sequences including three mutated nucleotides, which were confirmed by sequencing. (**D, E**) The host shutoff activities induced by rSY72 or rFX38 and their PA-X-deficient viruses at 12 and 24 hpi. The ribopuromycylation assay was conducted as described in the legend for Fig. 1A. The presence of PA-X, PA, NP, and GAPDH in cell lysates was analyzed by western blot using their corresponding antibodies. (**F, G**) The impact of SY72 or FX38 and their PA-X-deficient viruses on exogenous Rluc mRNA levels and luciferase activity. HET293T cells were transfected with the plasmid pRL-TK for 24 h and then infected with rSY72, rFX38, rSY72PA-FS, or rFX38PA-FS virus at an MOI of 1. At 12 and 24 hpi, the total cellular RNA was extracted, Rluc mRNA levels were measured by RT-qPCR, and the relative Rluc activity was determined with a GloMax 96 microplate luminometer. Data are shown as mean ± SD. ***, *P* < 0.001；****, *P* < 0.0001.

PA-X is the product of ribosomal frameshift (FS) of a highly conserved motif UCC UUU CGU, which is also part of the open reading frame of PA. To investigate the effect of PA-X knockdown expression on its host shutoff activity, we rescued the recombinant rSY72PA-FS and rFX38PA-FS viruses with PA-X-deficient expression in the rSY72 and rFX38 backgrounds, respectively, according to the scheme shown in Fig. 2A through C. A549 cells were infected with the rSY72, rFX38, and their corresponding PA-X defective viruses at an MOI of 1. The newly synthesized protein was detected at 12 and 24 hpi using a ribopuromycylation assay. As shown in Fig. 2D, the inhibitory effect on cellular protein synthesis efficiency triggered by rSY72PA-FS infection was significantly reduced at 24 hpi, compared with rSY72 infection. There was no significant difference in cellular protein synthesis efficiency between the rFX38PA-FS- and rFX38-infected cells (Fig. 2E). To investigate PA-X expression and whether the PA-X deficiency affects the expression of other viral proteins in the PA-X defective viruses, we determined expression levels of PA-X, PA, and NP by western blot. As shown in Fig. 2D and E, in cells infected with rSY72PA-FS, the expression level of PA-X was 41% of that observed in cells infected with rSY72. In cells infected with rFX38PA-FS, the expression level of PA-X was 55% of that observed in cells infected with rFX38PA. By contrast, the expression levels of PA were comparable between cells infected with rSY72 and those infected with rSY72PA-FS, as well as between cells infected with rFX38 and those infected with rFX38PA-FS. Similar results were also observed in the expression levels of NP. To further verify the inhibition of PA-X protein on host protein synthesis, mRNA levels and the fluorescence intensity of Rluc were detected by RT-qPCR and a dual luciferase reporter assay during infection with rSY72, rFX38, and their PA-X deficient viruses. As shown in Fig. 2F and G, mRNA levels (at 12 and 24 hpi) and fluorescence intensity of Rluc (at 24 hpi) were significantly enhanced in rSY72PA-FS-infected cells, compared with rSY72-infected cells. The mRNA levels and fluorescence intensity of Rluc in rFX38PA-FS-infected cells were comparable with those in rFX38-infected cells. These results indicated that knockdown of PA-X expression results in a remarkable reduction in host shutoff activity, especially in the background of rSY72 virus.

### Deficient expression of PA-X does not affect viral polymerase activity or replication capacity

**Fig 3:**
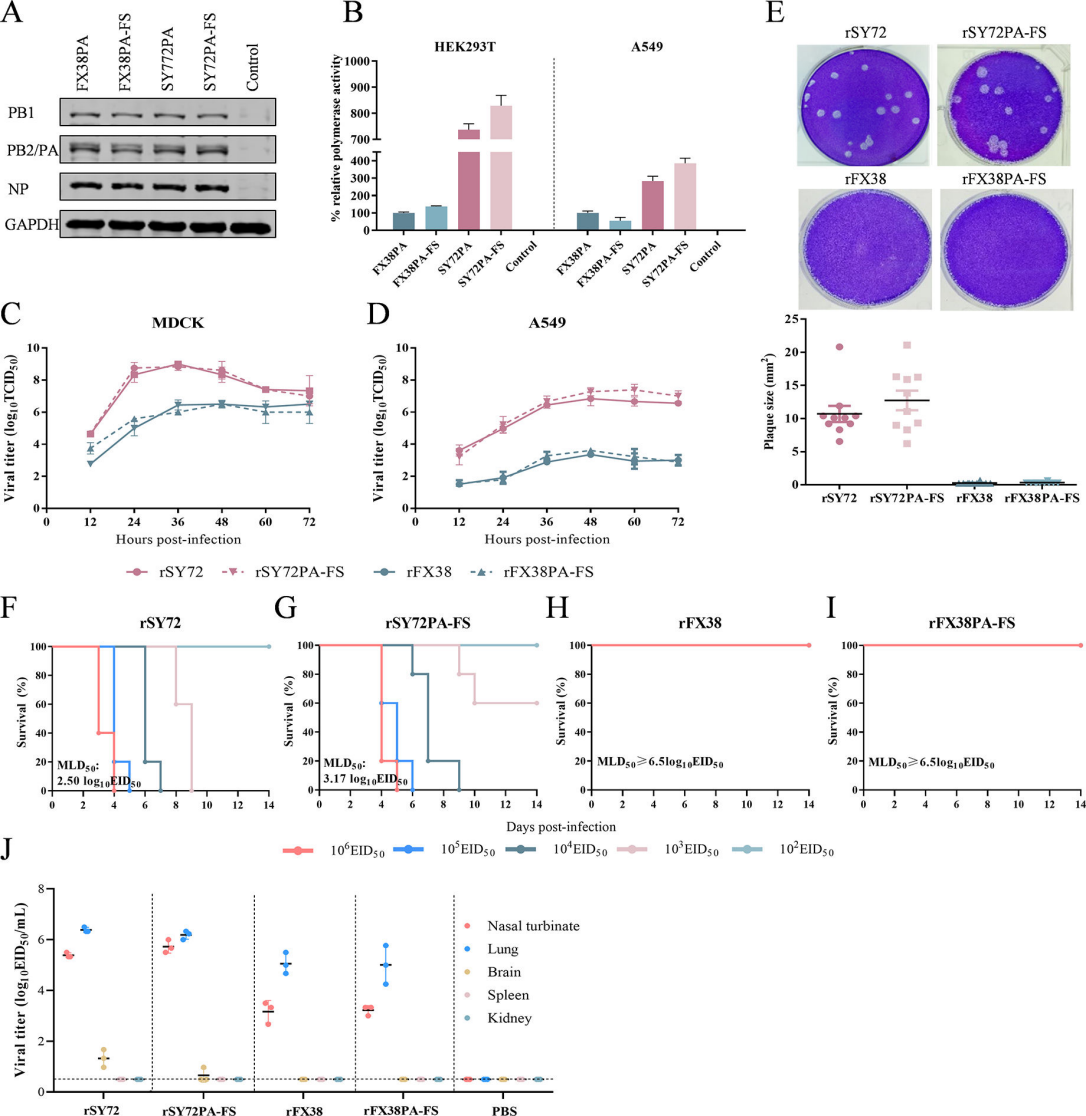
Effects of the PA-X-deficient on the viral polymerase activity, replication, and virulence of the rescued viruses. (**A, B**) Expression of the viral RNP components in HEK293T cells (**A**) and polymerase activity of the viral RNP in HEK293T or A549 cells (**B**). Cells were transfected with luciferase reporter plasmid (pPolI-Luc) and internal control plasmid *Renilla* (pRL-TK), together with plasmids expressing PB2, PB1, NP, and wild-type or mutant PA of SY72 or FX38 virus. At 24 hpi, HEK293T cell lysates were used to measure luciferase activities and detect expression of the indicated viral proteins. Polymerase activity was normalized to that of the wild-type vRNP of FX38. Values shown are the mean ± SD of three independent experiments. (**C, D**) Replication curves of rSY72, rFX38, and their respective PA-X-defective viruses. MDCK and A549 cells were inoculated at an MOI of 0.001 and 0.1, respectively. Virus titers were determined in MDCK cells at the indicated time points. Data are shown as the mean ± SD of three independent infections. (**E**) Plaque assay of the rSY72, rFX38, and their respective PA-X-defective viruses. Viruses were titrated under a 1% agarose overlay to ascertain the plaque phenotype. After 72 h, cells were fixed and stained with 0.5% crystal violet solution and plates were imaged (upper panel). Subsequently, the captured images were analyzed using ImageJ software to calculate the diameter of the plaques (lower panel). (**F–I**) MLD_50_ values of the rSY72 and rFX38 viruses and their PA-X-defective viruses. Five mice per group were intranasally inoculated with 10^2^, 10^3^, 10^4^, 10^5^, or 10^6^ EID_50_ (in a 50 μL volume) of the indicated viruses. Survival was monitored daily for 14 days after infection. Mice that lost ≥25% of baseline body weight were euthanized. The MLD_50_ values were calculated by the Reed–Muench method and expressed as log_10_ EID_50_. (**J**) Viral replication in mice. Groups of three 6-week-old female BALB/c mice were intranasally infected with 10^6^ EID_50_ of the indicated viruses. On day 3 p.i., three mice of each group were euthanized to collect organs for virus titration in eggs. Viral loads are expressed as the means ± SD.

To investigate whether deficient expression of PA-X affects viral polymerase activity, HEK293T cells were co-transfected with recombinant plasmids encoding PB2, PB1, NP, and wild-type PA or its PA-FS of SY72 and FX38 viruses. We determined the expressions of PB2, PB1, PA, and NP by western blot and found that deficient expression of PA-X had no obvious effect on expression levels of viral ribonucleoprotein (RNP) complex in cells (Fig. 3A). We further compared polymerase activity of the reconstituted RNP containing PA-FS and that of wild-type RNP of SY72 or FX38 in HEK293T and A549 cells using a mini-genome assay. As shown in Fig. 3B, compared to wild-type RNP, PA-FS protein had no detectable effect on polymerase activities of the reconstituted RNP of SY72 or FX38 in HEK293T cells. Similar results were also observed in A549 cells. Meanwhile, we tested the viral replication ability of rSY72, rFX38, and its PA-X-deficient viruses in MDCK and A549 cells. As shown in Fig. 3C and D, the viral titers of the PA-X-deficient viruses were comparable with those of the parental viruses at all time points in MDCK and A549 cells. We also compared the plaque sizes and found that the rSY72 and rSY72PA-FS viruses showed similar plaque-forming ability, whereas the rFX38 and rFX38PA-FS viruses failed to form detectable plaques in MDCK cells (Fig. 3E). We further tested the replication capacity and virulence of the parental virus and its PA-X-deficient viruses in BALB/c mice by determination of the MLD_50_. As shown in Fig. 3F and G, compared with rSY72 virus, the virulence of rSY72PA-FS virus was slightly decreased, with the MLD_50_ increasing from 2.5 log_10_ EID_50_ to 3.17 log_10_ EID_50_. The virulence of rFX38PA-FS was similar to that of rFX38 virus, both with MLD_50_ values above 6.5 log_10_ EID_50_ (Fig. 3H and I). Furthermore, the replication titers in the nasal turbinates, lungs, or the other organs of mice infected with the parental viruses and their PA-X-deficient viruses were comparable, indicating that the loss of PA-X protein expression had no detectable effects on viral replication in mice (Fig. 3J). Taken together, these data suggested that deficient expression of the PA-X protein slightly attenuates viral virulence in mice, but it has no significant effect on viral polymerase activity or replication capacity.

### PA-X with shutoff activity affects the host innate immune response

**Fig 4:**
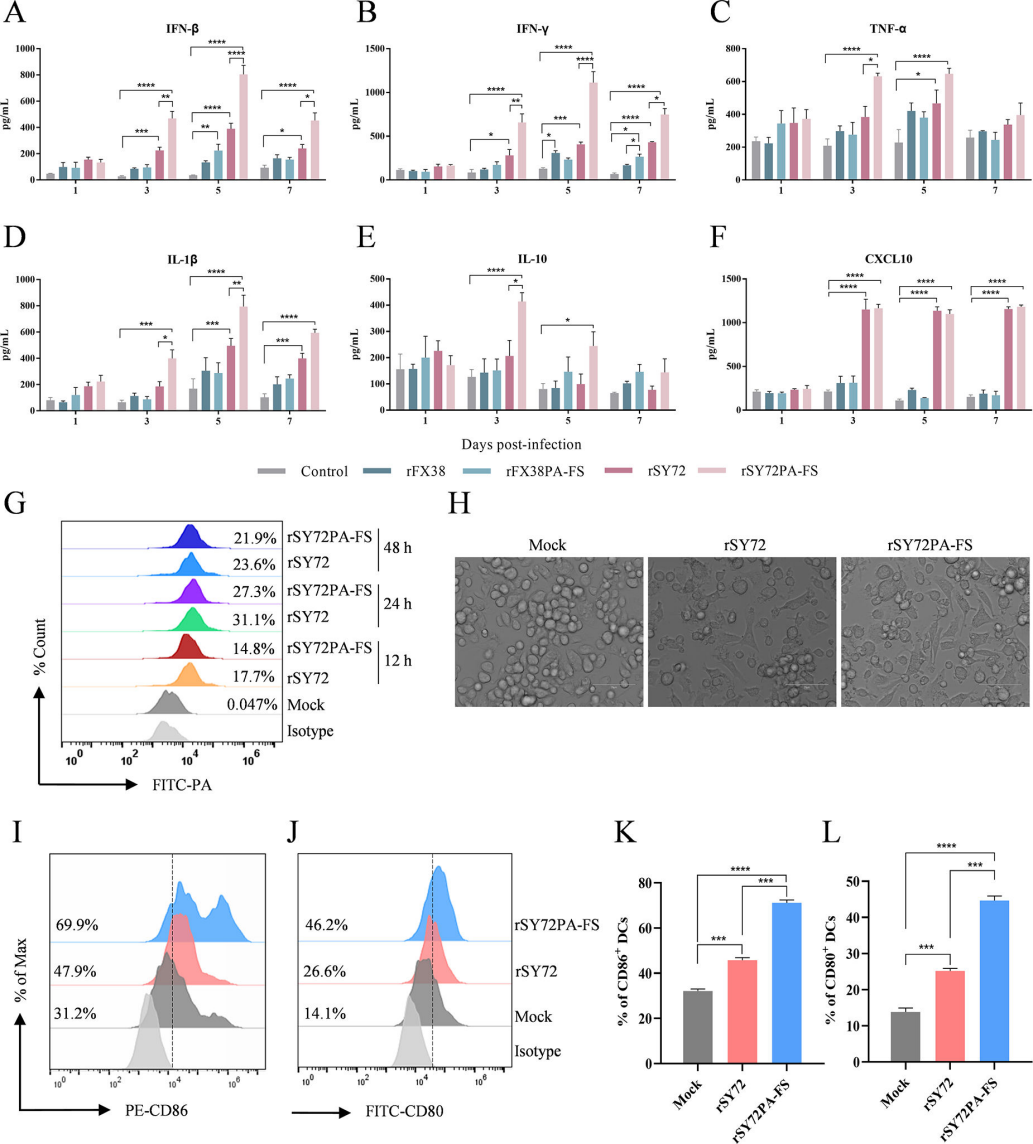
Assessment of innate immune responses induced by the viruses possessing wild-type and defective PA-X. (**A–F**) Effect of rSY72, rFX38, or PA-X-deficient virus on cytokine gene expression in the lungs of the mice. Groups of three 6-week-old female BALB/c mice were intranasally infected with 10^3^ EID_50_ of the recombinant viruses and the parental strains. The lungs of mice were collected at 1, 3, 5, and 7 dpi, and expression levels of the cytokines including IFN-β, IFN-γ, TNF-α, IL-1β, IL-10, and CXCL-10 were measured by ELISA. Data are the mean ± SD of three independent experiments. *, *P* < 0.05; **, *P* < 0.01; ***, *P* < 0.001; ****, *P* < 0.0001. (**G**) The infection levels of rSY72 and rSY72PA-FS in BM-DCs. BM-DCs were infected with rSY72 or rSY72PA-FS at an MOI of 1 and collected to detect PA protein expression (an index of viral replication) by flow cytometry. (**H**) Analysis of the morphological characteristics of BM-DCs. The colonies and dendrites of BM-DCs infected with rSY72 or rSY72PA-FS virus were observed using light microscopy, uninfected DCs were used as a control. Scale bar, 75 μm. (**I, J**) Phenotypic expression of BM-DCs. BM-DCs were infected with rSY72 or rSY72PA-FS at MOI of 1. At 24 hpi, the CD86 and CD80 expression were detected by flow cytometry. (**K, L**) Percentages of PE-CD86^+^ and FITC-80^+^ DCs were quantified by flow cytometry. Results were shown as the mean ± SD. ***, *P* < 0.001; ****, *P* < 0.0001.

PA-X protein may assist the virus in evading the immune response by reducing the expression of immune response-related cytokines through its host shutoff activity (33). To investigate the effect of PA-X with different host shutoff activities on the innate immune response, we intranasally inoculated mice with the rSY72 or rFX38 viruses, or their PA-X-deficient viruses at a dose of 10^3^ EID_50_. The lungs were collected from mice at 1, 3, 5, and 7 dpi, and the expression level of cytokines including interferon β (IFN-β), interferon γ (IFN-γ), tumor necrosis factor α (TNF-α), interleukin 1β (IL-1β), interleukin 10 (IL-10), and CXC motif chemokine ligand 10 (CXCL10) were detected by ELISA. As shown in Fig. 4A through F, higher expression levels of IFN-β, IFN-γ, TNF-α, IL-1β, IL-10, and CXCL10 in the lungs of mice infected with rSY72 and rSY72PA-FS viruses were detected since 3 dpi compared with those in the control. Notably, the expression levels of IFN-β and IFN-γ induced by rSY72PA-FS were significantly higher than those induced by rSY72 at 3, 5, and 7 dpi. Expression levels of TNF-α, IL-1β, and IL-10 activated by rSY72PA-FS were significantly higher than those induced by rSY72 at 3, 3 or 5, and 3 dpi, respectively. However, there was no significant difference in expression levels of CXCL10 induced by rSY72 and rSY72PA-FS at all time points. In contrast to rSY72 and rSY72PA-FS, both rFX38 and rFX38PA-FS failed to induce the production of cytokines other than IFN-β or IFN-γ, which was detected at 5 and 7 dpi. Compared to rFX38, rFX38PA-FS induced significantly higher expression level of IFN-γ at 7 dpi (Fig. 4B).

Dendritic cells (DCs) play a crucial role in antigen presentation and regulation of host innate immune responses. To further investigate how the PA-X protein of rSY72 regulates the innate immune function of DCs, we compared the infection abilities of rSY72 and rSY72PA-FS on murine BM-DCs by measuring PA protein expression at 12, 24, and 48 hpi. As shown in Fig. 4G, there was no significant difference in the infection abilities of the two viruses on DCs at the three time points, with infection peaking at 24 hpi. Additionally, the DCs stimulated by rSY72 and rSY72PA-FS viruses showed obvious dendritic structures at 24 hpi, compared to the un-stimulated DCs (Fig. 4H). CD86 and CD80 are important markers for evaluating the function and maturity of DCs (34). We detected the expression levels of these two markers on the surface of DCs after infection with rSY72 and rSY72PA-FS viruses by flow cytometry and found that the expression levels of CD86 and CD80 were both significantly increased at 24 hpi. In particular, the expression levels of CD86 and CD80 on the surface of DCs infected with rSY72PA-FS viruses were significantly higher than those on the rSY72-infected DCs (Fig. 4I through L). These results indicated that the PA-X protein possesses the ability to downregulate the phenotypic expression of DCs, and deficient expression of PA-X facilitates cytokine production in mice during influenza virus infection.

### The residue 122 in PA-X contributes to the host shutoff activity

**Fig 5:**
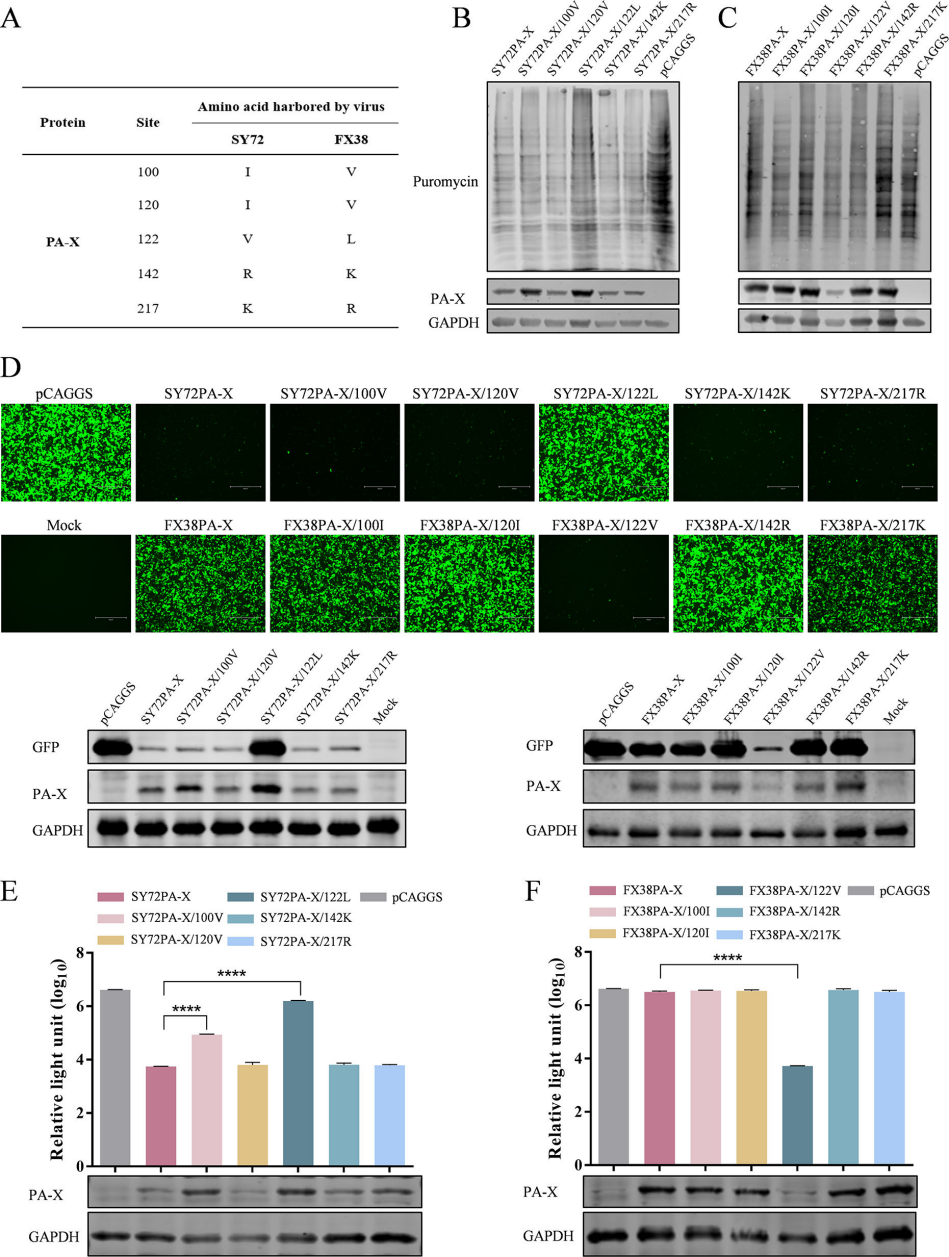
Identification of the amino acids affecting the shutoff activity of PA-X. (**A**) The amino acid differences in PA-X between the SY72 and FX38 viruses. (**B, C**) Assessment of the host shutoff activity induced by the indicated PA-X mutants. (**D**) The impact of PA-X mutants on exogenous GFP expression. (**E, F**) The impact of PA-X mutants on Rluc expression. These assays were conducted as described in the legend for Fig. 1, and the expression of GFP, PA-X, and GAPDH in cells was analyzed by western blot. Data are shown as mean ± SD from three independent experiments. ****, *P* < 0.0001.

The above studies showed that PA-X of SY72 and FX38 viruses induced significantly different host shutoff activity, resulting in different host immune response *in vivo*. PA-X sequence analysis indicated that the FX38 and SY72 viruses differed at five amino acids (positions 100, 120, 122, 142, and 217) (Fig. 5A). To identify amino acid residues responsible for the differences in host shutoff activity between the two viruses, we introduced single site-directed mutation into the PA-X gene of SY72 and FX38 viruses and evaluated the ability of the mutated PA-X to inhibit host protein synthesis in HEK293T cells. As shown in Fig. 5B, the inhibition of host protein synthesis induced by SY72PA-X/122L was significantly reduced compared with that induced by wild-type SY72PA-X. In contrast, the inhibition of host protein synthesis induced by the mutant SY72PA-X containing 100V, 120V, 142K, or 217R was comparable with that induced by SY72PA-X. When the reverse mutations were introduced into the FX38PA-X protein, the inhibition of host protein synthesis induced by the mutant FX38PA-X/122V was significantly enhanced compared with that induced by FX38PA-X (Fig. 5C). Furthermore, the inhibitory effects of PA-X protein on exogenous proteins were evaluated by measuring the expression of GFP and Rluc in HEK293T cells co-transfected with the plasmid pCAGGS encoding parental PA-X and its mutants. As shown in Fig. 5D, the SY72PA-X/122L mutant showed a significantly weaker inhibitory effect on GFP expression, whereas the FX38PA-X/122V mutant showed a significantly enhanced inhibitory effect. At 24 hpt, Rluc expression in cells transfected with pCAGGS encoding SY72PA-X/100V and SY72PA-X/122L was 14.7-fold and 285.2-fold, respectively, higher than that in SY72PA-X-transfected cells. The amino acid mutation at position 120, 142, or 217 did not significantly affect the inhibitory effect of SY72PA-X on exogenous gene expression (Fig. 5E). In contrast, Rluc expression in cells transfected with FX38PA-X/122V was 0.16% of that in cells transfected with FX38PA-X. However, the amino acid mutation at positions 100, 120, 142, or 217 showed no significant effect on the inhibitory effect of FX38PA-X on exogenous gene expression (Fig. 5F).

**Fig 6:**
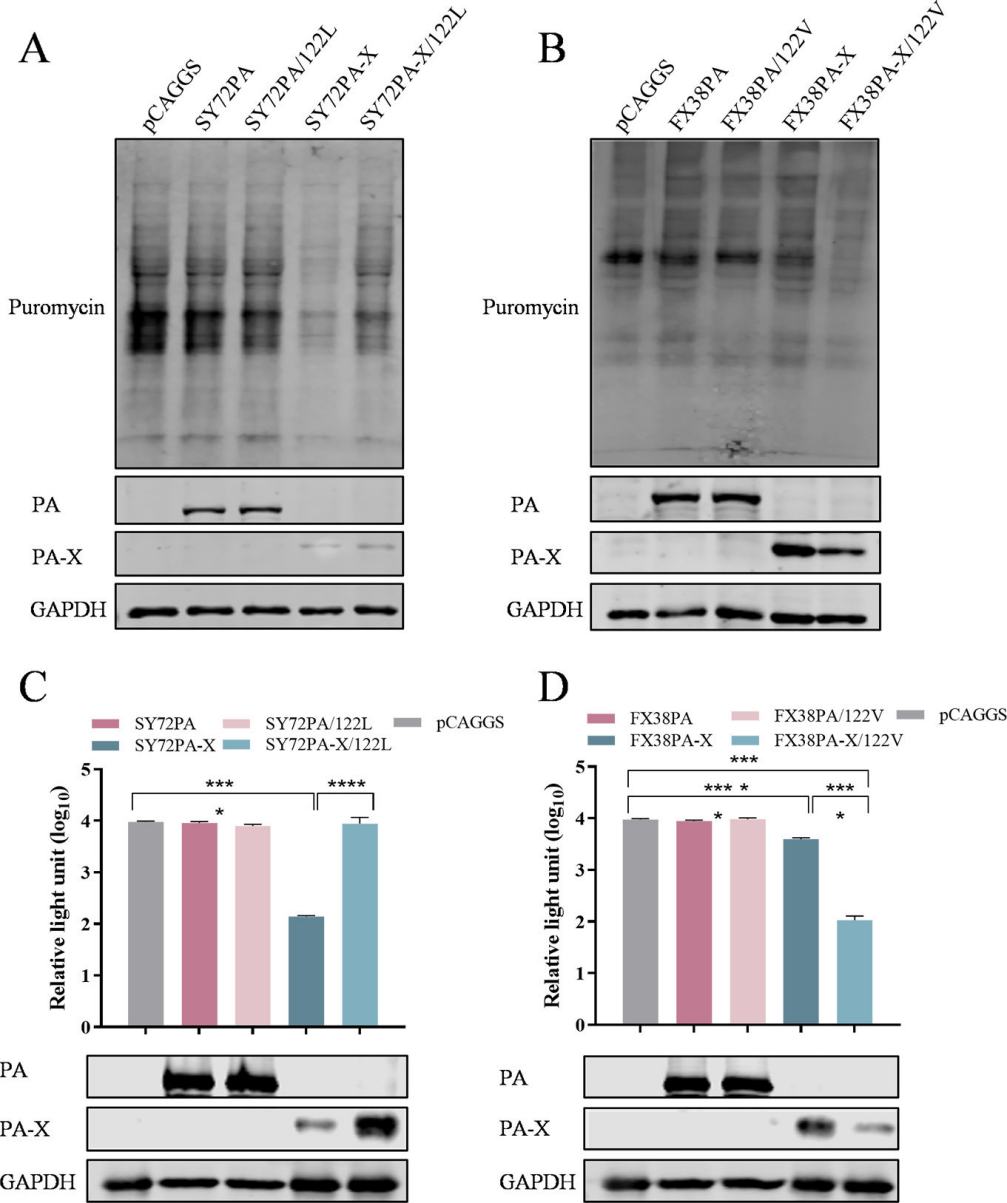
The residue 122V in PA-X contributes to its host shutoff activity. (**A, B**) Assessment of the host shutoff activity induced by wild-type PA, wild-type PA-X, and their respective mutants. (**C, D**) The impact of wild-type PA, wild-type PA-X, and their respective mutants on Rluc expression. These assays were conducted as described in the legend for Fig. 1. Data are shown as mean ± SD from three independent experiments. ****, *P* < 0.0001.

The N-terminus of the first 191 amino acids shared by PA-X and PA includes an RNA endonuclease domain that is required for host shutoff activity (9). Here, to distinguish the effect of 122V on PA-X and PA, we compared the inhibitory effects on cellular protein synthesis and ectopic Rluc expression of the wild-type PA-X, PA, and their mutants carrying V122L or L122V. As shown in Fig. 6A through D, no inhibitory effect on cellular protein synthesis or Rluc expression level was observed in cells transfected with the plasmid encoding wild-type PA of SY72 and FX38 or its respective mutant. However, the wild-type SY72PA-X and FX38PA-X exhibited a significant strong inhibitory effect on cellular protein synthesis and Rluc expression. Notably, a residue mutation of V122L drastically reduced the ability of SY72PA-X to inhibit host gene expression, while L122V significantly enhanced the ability of SY38PA-X to inhibit host gene expression (Fig. 6A through D). Taken together, these results demonstrated that a single amino acid at position 122 in the PA protein is crucial for the ability of PA-X to inhibit host gene expression, and the difference in host shutoff activity between SY72 and FX38 arises from residue 122 in PA-X, rather than in PA.

### The residue 122V in PA-X facilitates its suppression of host innate immune responses

**Fig 7:**
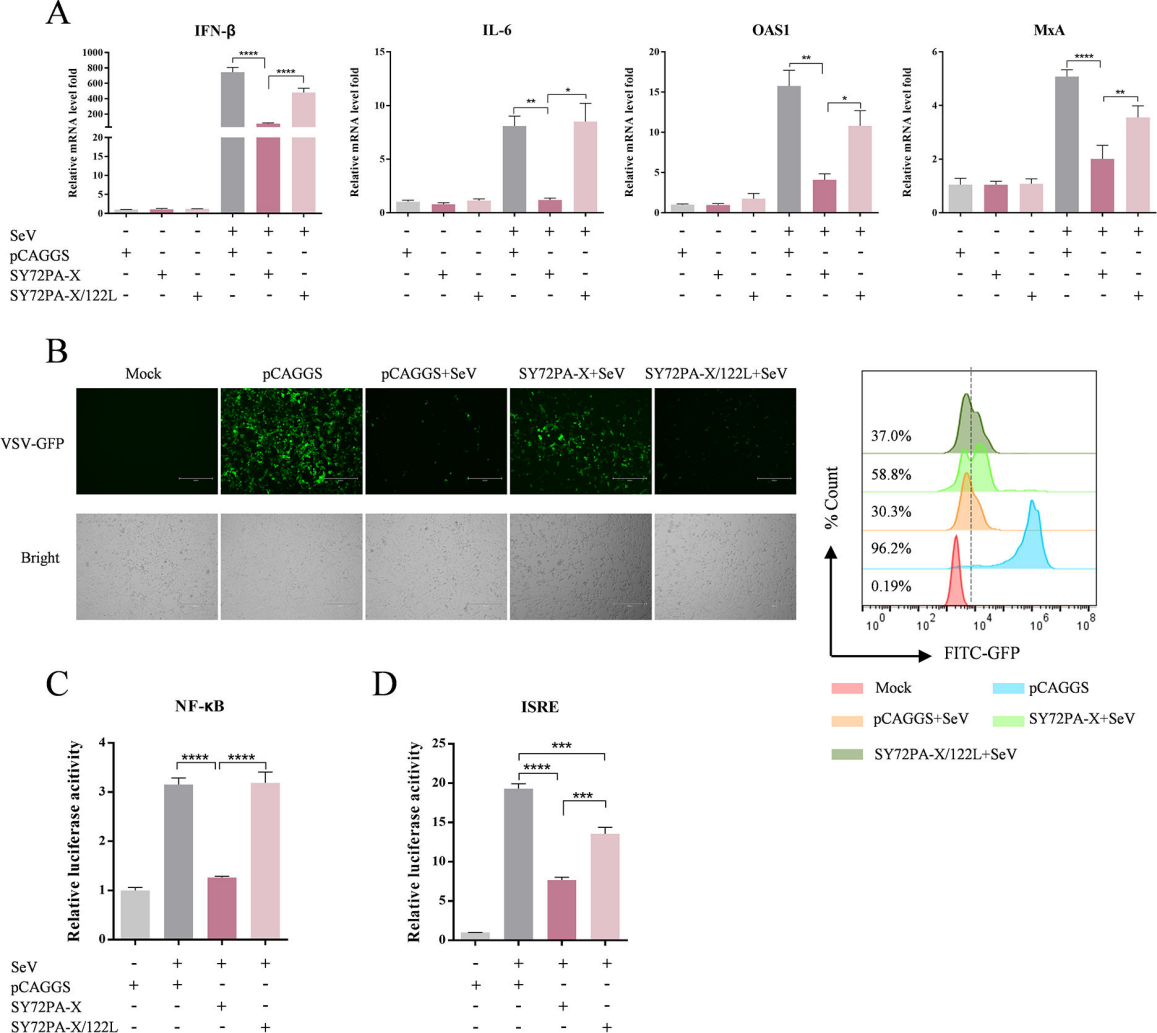
The residue 122V in PA-X contributes to regulating the innate immune responses induced by SeV infection. (**A**) Effects of SY72PA-X and SY72PA-X/122L on innate immune responses induced by SeV infection. HEK293T cells transfected with the plasmid encoding SY72PA-X or SY72PA-X/122L were infected with SeV at an MOI of 3. At 12 hpi, the IFN-β, IL-6, OAS1, and MxA expression levels were detected by RT-qPCR. (**B**) Inhibition of PA-X on SeV-induced antiviral effects. HEK293T cells were transfected with the plasmid encoding SY72PA-X or SY72PA-X/122L and infected with SeV at 24 hpt. After 12 h, cell supernatants were harvested and treated with UV light. HEK293T cells were incubated with the UV-treated supernatants and infected with VSV-GFP at an MOI of 1. At 12 hpi, GFP expression was evaluated by fluorescence microscopy (left panel) and quantified by flow cytometry (right panel). (**C, D**) Effect of PA-X on SeV-induced IFN and NF-κB signaling pathways. HEK293T cells were co-transfected with SY72PA-X or SY72PA-X/122L, together with pRL-TK, and pISRE-Luc or pNF-κB-Luc, followed by infection with SeV at 24 hpi. The luciferase activity was measured at 12 hpi. The transfection efficiency was normalized by calculating the ratio of firefly luciferase activity to *Renilla* luciferase activity, and the results were expressed relative to empty plasmid controls (-SeV). Data are shown as the mean ± SD of three independent experiments. *, *P* < 0.05; **, *P* < 0.01; ***, *P* < 0.001; ****, *P* < 0.0001.

To determine the effect of residue 122 in the PA-X protein on the innate immune response, we measured the mRNA levels of innate immune cytokines in cells transfected with the plasmid encoding SY72PA-X or SY72PA-X/122L, followed by SeV infection. As shown in Fig. 7A, the mRNA levels of IFN-β, interleukin 6 (IL-6), 2'-5'-oligoadenylate synthetase 1 (OAS1), and myxovirus resistance protein A (MxA) in cells transfected with the plasmid encoding SY72PA-X were significantly lower than those in cells transfected with empty plasmid. By contrast, the mRNA levels of all four immune cytokines were significantly enhanced in cells transfected with SY72PA-X/122L compared with those in cells transfected with SY72PA-X. By measuring the replication of VSV-GFP in HEK293T cells, as evaluated by GFP expression and quantified using flow cytometry, we further investigated the functional role of PA-X in antiviral immune responses. As shown in Fig. 7B, VSV-GFP replication was drastically inhibited by the cell supernatant transfected with empty plasmid followed by SeV infection. When HEK293T cells were transfected with the plasmid encoding SY72PA-X prior to SeV infection, the ability of their supernatant to inhibit VSV-GFP replication was significantly reduced. By contrast, HEK293T cells were transfected with the plasmid encoding SY72PA-X/122L prior to SeV infection, the ability of their supernatant to inhibit VSV-GFP replication was significantly enhanced.

To investigate the inhibitory effect of the SY72PA-X protein on innate immune signaling, the effects of the SY72PA-X protein on the IFN and NF-κB responses induced by SeV infection were evaluated by a dual luciferase reporter assay. We found that the SY72PA-X protein significantly inhibited the activation of the ISRE and NF-κB promoters following SeV infection, whereas the inhibitory effects of the SY72PA-X/122L protein on the activation of the ISRE and NF-κB promoters were significantly reduced compared to the SY72PA-X protein (Fig. 7C and D). These results demonstrated that PA-X protein inhibits the secretion of antiviral factors stimulated by SeV, and the residue 122V in PA-X facilitates its suppression of host innate immune responses.

### The residue 122V in PA-X plays a crucial role in suppressing host gene expression across various influenza virus strains

To investigate the polymorphism of the amino acid residue 122 in PA-X, the available PA-X gene sequences of H1, H3, H5, H7, and H9 influenza viruses isolated from swine, avian, and human samples, irrespective of NA subtype, were downloaded from the NCBI Influenza Virus Resource (https://www.ncbi.nlm.nih.gov/genomes/FLU/Database/nph-select.cgi?go=database; up to 11 March 2025). Alignments of the amino acid sequences of the corresponding regions of the PA-X proteins were conducted using the MAFFT (Multiple Alignment using Fast Fourier Transform) program (https://mafft.cbrc.jp/alignment/software/). As shown in Table 1, the residue 122V in PA-X was highly conserved among all of the different subtypes of influenza viruses, irrespective of host origin. The residue 122I was also occasionally detected in H1, H3, H5, and H9 influenza viruses. By contrast, residues 122L, 122A, and 122E were individually detected in H1N1 (FX38), H3N8 (A/northern pintail/Alaska/AK19-534/2019), and H5N1 (A/black vulture/Georgia/W22-723A/2022) viruses, respectively.

**TABLE 1:** Polymorphism frequencies of amino acid residue 122 in the PA-X protein of different subtypes of influenza viruses^a^

Virus subtype	Host (n)	Prevalence (%) of the indicated amino acid				
		V	I	L	A	E
H1	Swine (2765)	99.2 (2744)	0.7 (20)	0.04 (1)		
	Avian (251)	99.2 (249)	0.8 (2)			
	Human (930)	99.8 (928)	0.2 (2)			
H3	Swine (1050)	99.3 (1043)	0.7 (7)			
	Avian (567)	99.1 (562)	0.7 (4)		0.2 (1)	
	Human (1697)	100 (1697)				
H5	Swine (7)	100 (7)				
	Avian (1168)	98.8 (1154)	1.1 (13)			0.1 (1)
	Human (17)	100 (17)				
H7	Swine (1)	100 (1)				
	Avian (516)	100 (516)				
	Human (42)	100 (42)				
H9	Swine (5)	100 (5)				
	Avian (957)	98.5 (943)	1.5 (14)			
	Human (3)	100 (3)				

^a^ The amino acid frequencies were determined for unique PA-X full-length sequences downloaded from the NCBI Influenza Virus Resource (https://www.ncbi.nlm.nih.gov/genomes/FLU/Database/nph-select.cgi?go=database). n, number of sequences analyzed.

**Fig 8:**
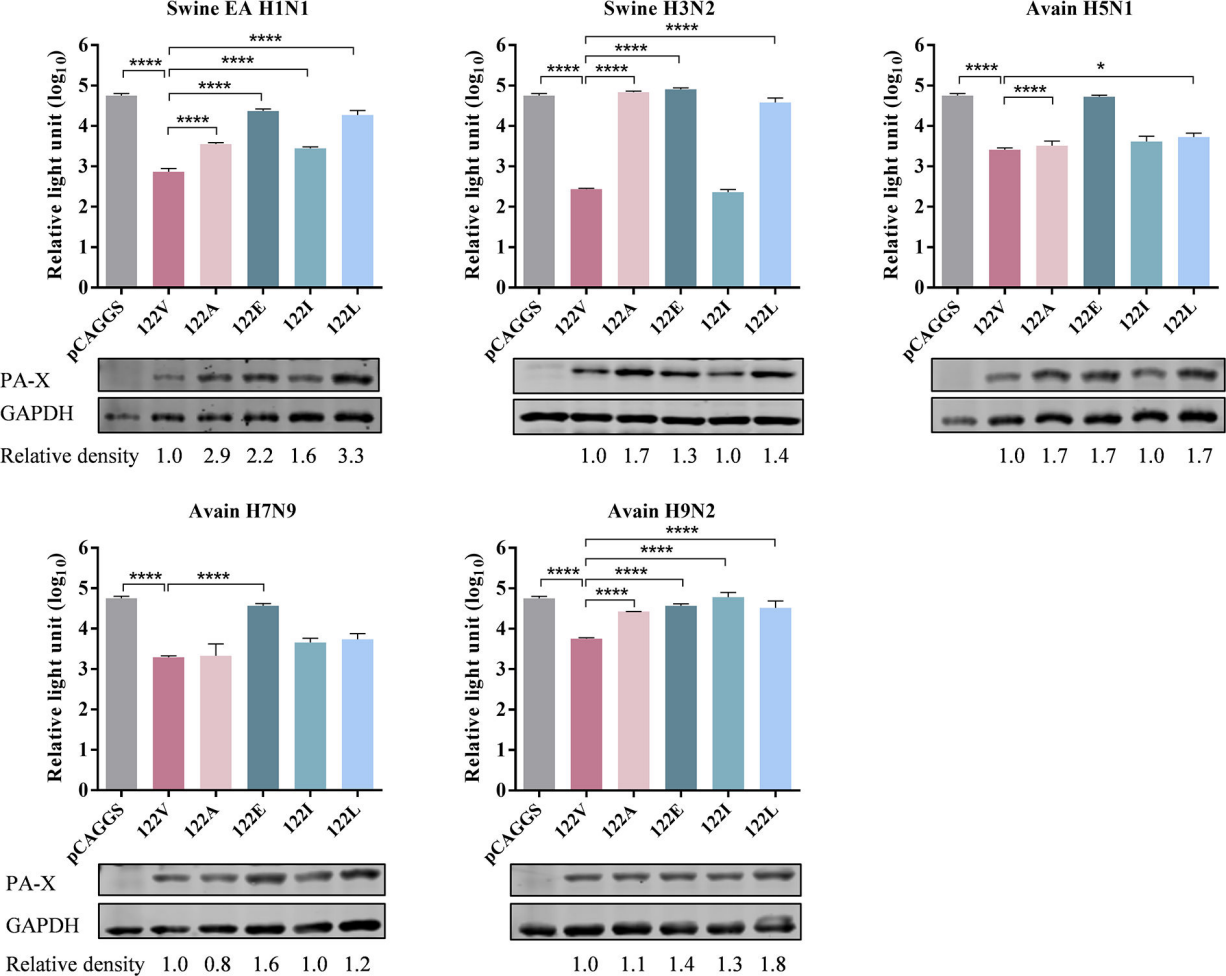
Impacts of the residue 122 in PA-X on its suppression of Rluc expression in multiple subtypes of influenza viruses. HEK293T cells were co-transfected with pRL-TK and the recombinant pCAGGS plasmid encoding the wild-type or mutant PA-X. Luciferase expression was determined as described in the legend for Fig. 1C. Data are presented as the mean ± SD of three biological replicates. *, *P* < 0.05; ***, *P* < 0.001; ****, *P* < 0.0001, compared to wild-type PA-X.

To evaluate the effect of residue 122 detected in PA-X on its host shutoff activity across various influenza virus strains, five wild-type PA-X harboring 122V from swine EA H1N1, swine H3N2, avian H5N1, avian H7N9, and avian H9N2 viruses were selected for PA-X mutant construction by introducing single site-directed mutation V122A, V122E, V122I, or V122L. The inhibitory effects of PA-X protein on Rluc expression were determined in HEK293T cells co-transfected with pRL-TK and the plasmid pCAGGS encoding wild-type PA-X or its mutants. As shown in Fig. 8, the wild-type PA-X of all tested viruses exhibited strong inhibitory effects on Rluc expression. Compared to wild-type PA-X with 122V, all four PA-X mutants harboring 122A, 122E, 122I, and 122L of swine EA H1N1 or avian H9N2 viruses showed significantly decreased inhibitory effects on Rluc expression. In contrast, three PA-X mutants of swine H3N2 virus (122A, 122E, and 122L), two of avian H5N1 virus (122E and 122L), and one PA-X mutant of avian H7N9 virus (122E) exhibited significantly reduced inhibitory effects on Rluc expression compared to wild-type PA-X (Fig. 8). Furthermore, we determined the PA-X expression level in HEK293T cells transfected with the indicated plasmids by western blot analysis. Compared to wild-type PA-X with 122V, PA-X mutants carrying different residue 122 showed diverse expression levels in cells, which highlighted that different residues in PA-X displayed varying effects on its host shutoff function and also exerted differential effects on its expression in host cells (Fig. 8). These results indicated that PA-X 122V is highly conserved and plays a crucial role in suppressing host gene expression across various influenza virus strains.

## DISCUSSION

PA-X, an accessory protein encoding by the PA gene of influenza A virus, modulates viral pathogenicity and virus-induced host innate immune responses via suppression of host gene expression or cellular shutoff, through cellular mRNA cleavage (28, 35). Our previous study has demonstrated that PA contributes to virulence differences of the two genetically similar EA H1N1 viruses (SY72 and FX38). In this study, we determined the host shutoff activities induced by these two viruses and found that SY72 and FX38 infections displayed significantly different inhibitory effects on host gene expression. Both PA-X and NS1 are key factors for influenza virus to assist in viral immune escape by inhibiting host gene expression during infection, and their cooperative relationship varies among different virus strains (36–38). For example, in the A/PR8/34 (H1N1) virus, the function of the NS1 effector domain enhances the host shutoff activity of PA-X (39), while in the 2009/H1N1 virus, the inhibitory activity of PA-X on co-expressed proteins is stronger than that of NS1 (40). Here, by measuring the expression levels of endogenous and exogenous proteins in cells, we determined that the PA-X protein plays a dominant role in regulating the host shutoff activity, rather than the NS1 protein. This was consistent with a previous finding that only PA-X protein, not NS1 protein, had a significant inhibitory effect in the 2009/H1N1 virus (41). We speculate the reason is that the PA genes of SY72 and FX38, which are genetically derived from the 2009/H1N1 virus, to some extent maintain the predominant role of PA-X in the host shutoff function of these reassortant EA H1N1 influenza viruses.

Expression efficiency of PA-X protein can modulate viral polymerase activity, potentially influencing viral replication, virulence, and transmissibility in mammalian and avian hosts. In this study, we generated two recombinant viruses carrying deficient PA-X protein of SY72 and FX38 and found that loss of PA-X expression resulted in significant reduction in its host shutoff activity, especially in the background of rSY72 virus. We further compared their viral polymerase activity, replication capacity, and virulence in mice. Compared to the rSY72 virus, PA-X-deficient expression slightly decreased the virulence of rSY72PA-FS in mice. However, no significant difference in viral polymerase activity or replication capacity was observed between rSY72 and rSY72PA-FS, or between rFX38 and rFX38PA-FS. These findings highlight the fact that although the host shutoff activity of PA-X exists in all subtypes of influenza A viruses, the regulation results of shutoff activity on virulence and replication capacity are different or even opposite among the various subtypes (14, 15, 42–47). Even for the same subtype of influenza viruses, inconsistent results were observed in the independent studies (15, 43, 48). Therefore, we supposed that some factors other than the virus itself also participate in modulating the viral replication and virulence in different host species.

The PA-X protein can effectively modulate the antiviral innate immune response by suppressing host protein synthesis, thereby promoting efficient viral replication and transmission. Herein, we measured expression levels of the innate immune factors including IFN-β, IFN-γ, TNF-α, IL-1β, IL-10, and CXCL-10 in the lungs of mice infected with the parental viruses and their PA-X-deficient viruses and found that significantly stronger innate immune responses were induced by the rSY72PA-FS virus than by the rSY72 virus. DCs are immune cells that can secrete various cytokines and present antigens to T cells (49). In this study, we further investigated the effects of rSY72 and rSY72PA-FS on immune cell function and found that although rSY72 and rSY72PA-FS exhibited comparable infection kinetics in murine BM-DCs, rSY72PA-FS could upregulate the expression levels of phenotypic markers such as CD86 and CD80 on immature DCs compared to rSY72. Therefore, we concluded that the downregulation of DC activation markers by PA-X may represent a viral strategy to evade early innate immune detection, thereby reinforcing the notion that PA-X exerts an immunosuppressive effect in the context of influenza virus infection. Of note, our present study evaluated the bilateral effects of PA-X-deficient expression and demonstrated that although the viral polymerase activity and replication ability were not significantly impacted by the reduced host shutoff activity of defective PA-X of SY72. The enhanced innate immune responses induced by rSY72PA-FS still attenuated the virulence phenotype in the mouse model, albeit to some extent. This finding highlights the important role of PA-X’s host shutoff activity in regulating viral fitness and virulence of influenza A viruses (50–52).

Given the significantly different virulence phenotypes of the SY72 and FX38 viruses, we have identified two amino acids at positions 100 and 122 in the PA protein that are key virulence determinants (30). Both of these amino acids are located in the 191-amino acid N-terminal domain shared by the PA and PA-X protein, which has endonuclease activity to inhibit host protein synthesis. Our present study demonstrated that the significant difference in host shutoff activity arises from PA-X, not PA. We pinpointed a single amino acid, 122V, in the N-terminal region of PA-X responsible for the higher host shutoff activity of the SY72 virus, which is consistent with the previous findings that the endonuclease active sites and residues around the endonuclease active sites of PA-X are important for its host shutoff activity (9, 40, 53–55). During virus infection, antiviral innate immune responses are initiated in the host cells, leading to the production of type I IFN and NF-κB-dependent cytokines (56, 57). In this study, by determining the effects of PA-X on production of innate immune cytokines, we found that PA-X plays a crucial role in suppressing the host innate immune response, and the PA-X with 122V exhibits significantly stronger suppression ability than PA-X with 122L. We further demonstrated that PA-X with 122V displays significantly stronger inhibitory effects on the activation of ISRE or NF-κB promoters compared to PA-X with 122L. Our data in this study revealed that the ability of PA-X to suppress the host innate immune responses is closely related to its host shutoff activity.

Adaptation is considered to drive evolution by conferring mutations that enhance fitness and natural selection, which is an important mechanism for cross-species transmission of influenza viruses (58). Our present and previous studies have found that the amino acid at position 122 in the PA-X protein (same position in PA) is highly conserved across multiple subtypes of influenza viruses, suggesting that this residue plays an important dual role in polymerase function and host shutoff activity. Residue 122V in the PA protein has been found to be closely correlated with higher polymerase activity, viral replication capacity, and virulence in mice (30), which may contribute to elevating viral fitness both *in vitro* and *in vivo*. In this study, we further demonstrated the crucial role of PA-X 122V in determining the host shutoff activity across multiple subtypes of influenza viruses. In particular, we found that in some viruses, any mutations at position 122 in PA-X result in significantly decreased host shutoff activity. This adaptation may reflect a trade-off between immune evasion and viral fitness. One possible explanation for the high conservation of PA-X 122V is that the impaired host shutoff activity may present disadvantages for viral replication across multiple hosts, thereby restricting natural selection of influenza virus variants harboring an amino acid substitution at position 122 in PA-X.

In summary, our findings demonstrate the crucial role of the PA-X protein in suppressing host protein synthesis and further emphasize the importance of PA-X protein in counteracting the host’s innate immune response to influenza A virus. Moreover, we identified a novel residue, 122V, in the N-terminal region of PA-X responsible for host shutoff activity, thereby affecting the ability of virus to inhibit host antiviral innate immune responses. These findings broaden the understanding of the function and potential roles of PA-X or PA in the pathogenicity of influenza A virus. Further study is needed to explore the intrinsic mechanism underlying the enhanced host shutoff activity mediated by PA-X 122V and its subsequent contribution to regulating viral pathogenesis across the different hosts.

## MATERIALS AND METHODS

### Cells, viruses, and plasmids

Madin-Darby canine kidney (MDCK), human embryonic kidney (HEK293T), and Verda Reno (Vero) cells were maintained in Dulbecco’s modified Eagle’s medium (DMEM) (Gibco, Grand Island, NY, USA) supplemented with 5%, 10%, and 10% fetal bovine serum (FBS) (Gibco), respectively. Human lung carcinoma cells (A549) were maintained in Ham’s F12 medium (Gibco) supplemented with 10% FBS. HEK293T stably expressing enhanced green fluorescent protein (HEK293T-EGFP) cells were constructed as described previously (59) and maintained in DMEM supplemented with 10% FBS. Dendritic cells (DCs) were prepared from murine bone marrow (BM) cells and maintained in RPMI-1640 supplemented with 10% FBS, 100 U/mL penicillin G sodium, 100 μg/mL streptomycin, 20 ng/mL IL-4, and 20 ng/mL GM-CSF.

Two wild-type EA H1N1 influenza viruses (FX38 and SY72) and their recombinant viruses (rFX38 and rSY72) were generated by reverse genetics as described previously (30). All viruses were propagated in 10-day-old specific pathogen-free (SPF) embryonated chicken eggs, followed by determining the 50% egg infectious dose (EID_50_). Sendai virus (SeV) was propagated in SPF chicken embryos, and the virus titer was determined by a hemagglutination test as described previously (60). Vesicular stomatitis virus expressing GFP (VSV-GFP) was propagated in Vero cells.

The pcDNA3.1 plasmids encoding PB1, PB2, NP, and wild-type PA of the SY72 and FX38 viruses were generated in our previous study (30). The pCAGGS plasmids encoding NS1 of SY72 and FX38, and wild-type or mutant PA-X of SY72, FX38, and the other subtypes of influenza viruses used in this study were constructed by inserting their corresponding ORF sequences in the pCAGGS. The integrity and accuracy of the indicated genes were verified by sequencing to ensure the absence of unwanted mutations. All sequences of the viral genes and primers are available upon request.

### Ribopuromycylation assay

Puromycin is a compound that binds newly translated polypeptides, which causes the termination of translation of the full-length proteins. To assess the ability of virus infection to inhibit host protein synthesis, we employed a ribopuromycylation assay using the anti-puromycin monoclonal antibody to directly monitor the level of puromycin. A549 cells were infected with the wild-type SY72 or FX38 virus and its mutant viruses. At 12 and 24 hpi, the cells were treated with puromycin (5 μg/mL) for 45 min and then subjected to western blot analysis. Experiments to evaluate the ability of PA-X and NS1 protein to inhibit host gene expression were carried out using 293T cells transfected with the corresponding pCAGGS-PA-X and pCAGGS-NS1 plasmids, respectively, at 24 hpt.

### GFP expression assay

HEK293T cells were co-transfected with a recombinant pCAGGS plasmid encoding wild-type PA-X, NS1, or PA-X mutants, and a pCAGGS plasmid encoding GFP. At 24 hpt, the cells were observed under a fluorescence microscopy and then re-suspended with PBS and the expression of GFP was detected by flow cytometry. The fluorescence intensity was standardized to the cells transfected with the pCAGGS empty vector.

### Single luciferase reporter assay

HEK293T cells were co-transfected with recombinant plasmids expressing wild-type PA-X, NS1, or PA-X mutants, along with the *Renilla* luciferase reporter plasmid (pRL-TK). At 24 hpt, cells were lysed with passive lysis buffer and measured using reagents in the Dual Luciferase Reporter Assay System Kit (Promega, Madison, WI, USA), according to the manufacturer’s instructions.

### Construction of PA-X-deficient virus

According to the method described previously (14), the PA gene X-ORF 5ʹ-UCCUUUCGU-3ʹ sequence of the rSY72 and rFX38 viruses was mutated to 5ʹ-UCCUU**CA**G**A**-3ʹ by site-directed mutagenesis using a point mutation kit (Vazyme, Nanjing, China) to knock down the expression of PA-X. Then, two recombinant viruses, designated rSY72PA-FS and rFX38PA-FS, containing the mutated PA gene in the rSY72 and rFX38 backgrounds, respectively, were rescued by transfection in HEK293T cells and identified by whole genome sequencing to ensure the absence of unwanted mutations. The primer sequences are available upon request.

### Virus replication kinetics

To evaluate the viral replication properties *in vitro*, confluent monolayers of MDCK and A549 cells were infected with the indicated viruses at a multiplicity of infection (MOI) of 0.001 and 0.1, respectively. After incubation for 1 h at 37°C, the viral inoculum was replaced with DMEM containing 1 µg/mL and Ham’s F-12K containing 0.25 µg/mL of tolylsulfonyl phenylalanyl chloromethyl ketone-treated trypsin (TPCK-trypsin) (Sigma-Aldrich, St. Louis, MO, USA), followed by further incubation at 37°C. Cell culture supernatants were collected at 12, 24, 36, 48, 60, and 72 hpi for virus titration in MDCK cells.

### Plaque assay

To evaluate the plaque characteristics induced by the viruses, a monolayer of confluent MDCK cells in 6-well plates was inoculated with the indicated viruses serially diluted from 10^5^ to 10 TCID_50_/mL. After 1 h of incubation, the infection media were removed and replaced with an agarose overlay containing 2×MEM with 0.6% BSA, 2 µg/mL TPCK-trypsin, and 2% SeaPlaque agarose (Lonza Group, Basel, Switzerland). The agarose overlay was removed at 72 hpi, and the cells were fixed with formaldehyde and stained with 0.5% crystal violet in methanol for 10 min. The stained plates were washed with water and dried before imaging. The sizes of 10 randomly selected plaques were measured using ImageJ software (https://imagej.net/ij/).

### Minigenome assay

A minigenome assay was performed to determine viral polymerase activity. Briefly, HEK293T or A549 cells were transfected with pcDNA3.1(+) plasmids encoding PB2, PB1, NP, and PA (the wild-type or mutant), together with a plasmid expressing the firefly luciferase gene under the control of RNA polymerase I promoter (pPolI-Luc), and pRL-TK as an internal control. Polymerase activity was calculated by standardization of the firefly luciferase activity to the *Renilla* luciferase activity.

### Pathogenicity study in mice

Groups of five 6-week-old female BALB/c mice (Vital River Laboratories, Beijing, China) were anesthetized with averdin (Sigma-Aldrich) and intranasally inoculated with 10^2^ to 10^6^ EID_50_ of the indicated viruses. Body weight and survival were monitored daily for 14 dpi. Mortality was recorded either as an actual death or body weight loss ≥25%, which is the threshold for humane euthanasia. To evaluate the viral replication capacity *in vivo*, three mice were intranasally inoculated with 10^6^ EID_50_ of the indicated viruses. At 3 dpi, the mice were euthanized and their organs, including the brain, nasal turbinate, lungs, spleen, and kidneys, were harvested and titrated in 10-day-old embryonated chicken eggs.

### Reverse-transcription quantitative polymerase chain reaction (RT-qPCR)

HEK293T cells transfected with the recombinant plasmids were infected with SeV at an MOI of 3, and the total RNA was extracted using RNAsimple Total RNA Kit (Tiangen, Beijing, China). Then, the corresponding cDNA was transcribed with oligo(dT) primers using HiScript II 1st Strand cDNA Synthesis Kit (Vazyme) and subjected to qPCR with 2×SYBR green PCR master mix (Vazyme) following the manufacturer’s instructions. The primers used in this study are available upon request. The expression level of each gene was normalized to expression of glyceraldehyde 3-phosphate dehydrogenase (GAPDH) as a control using the 2^−ΔΔCt^ method. Each experiment was performed in triplicate.

### Enzyme-linked immunosorbent assay (ELISA)

To characterize the innate immune response *in vivo*, groups of 12 6-week-old female BALB/c mice were intranasally inoculated with 10^3^ EID_50_ of rSY72, rFX38, and their PA-X-deficient mutants. At 1, 3, 5, and 7 dpi, three mice per group were euthanized and the lungs were collected for cytokine expression analysis. The expression levels of the cytokines in the lungs of virus-infected mice were detected using the mouse IFN-β, IFN-γ, TNF-α, IL-1β, IL-10, and CXCL10 ELISA kit (MLBio, Shanghai, China), according to the manufacturer’s instructions.

### Viral infectivity on murine BM-DCs and the maturity of DCs

DCs were infected with the rSY72 and rSY72PA-FS viruses at an MOI of 1. Uninfected cells were used as a negative control. The cells collected at 12, 24, and 48 hpi were fixed with 4% paraformaldehyde, permeabilized in 0.5% Triton X-100, and probed with monoclonal antibodies against PA protein and IRDye800CW goat anti-mouse IgG (H + L), then detected by flow cytometry. The cells collected at 24 hpi were incubated with PE-CD86 or FITC-CD80. The expression levels of DC phenotypic markers were detected by flow cytometry to evaluate the maturity of DCs.

### Inhibition of the antiviral effect by PA-X

HEK293T cells were first transfected with the pCAGGS plasmid encoding SY72-PA-X or SY72-PA-X/122L, and then infected with SeV at 24 hpt. Cell supernatants were harvested at 12 hpi and treated with UV light for 20 min. Then, the HEK293T cells were incubated with the UV-treated supernatants prior to infection with VSV-GFP. At 12 hpi, GFP expression was evaluated by fluorescence microscopy and quantified by flow cytometry.

### Western blotting

Cells were lysed in NP-40 lysis buffer (Beyotime, Shanghai, China) containing a protease inhibitor cocktail (Roche, Basel, Switzerland). Cellular lysate proteins were boiled with 5× sodium dodecyl sulfate (SDS) loading buffer (Beyotime) and separated using 10% SDS-polyacrylamide gel electrophoresis, then transferred onto nitrocellulose membrane. The membrane was incubated with 5% (wt/vol) skim milk and then probed with the indicated primary antibodies (the polyclonal or monoclonal antibody specific for viral protein, puromycin, GFP, and GAPDH) and the corresponding secondary antibody (IRDye 800CW goat anti-mouse or anti-rabbit IgG [H+L]). Finally, the blots were scanned using an Odyssey Infrared Imaging System (Li-Cor Biosciences, Lincoln, NE, USA) for further analysis.

### Dual luciferase reporter assay

HEK293T cells were transfected with pCAGGS plasmids encoding wild-type PA-X and its corresponding mutant PA-X, together with pRL-TK, and a plasmid expressing firefly luciferase under the control of the ISRE promoter (pISRE-Luc) or the NF-κB promoter (pNF-κB-Luc). At 24 hpt, the cells were stimulated with SeV for 12 h. The luciferase activities were measured using the Dual Luciferase Reporter Assay System Kit (Promega), according to the manufacturer’s instructions.

### Statistical analysis

Statistical significance was determined using Student’s *t*-test for two-group comparisons and one-way or two-way analysis of variance with Tukey’s post-test for multiple-group comparisons in GraphPad Prism version 8.0 (GraphPad Software Inc., CA, USA). A probability (*P*) value lower than 0.05 was considered statistically significant.
